# Editorial: Precision medical approach-driven multi-dimensional diagnosis and treatment strategies

**DOI:** 10.3389/fmed.2023.1164830

**Published:** 2023-03-21

**Authors:** Richard Beatson, Cong-Qiu Chu, Su Jong Yu, Xiaolong Liu, Jie Zhao

**Affiliations:** ^1^Respiratory Medicine, University College London, London, United Kingdom; ^2^Division of Arthritis and Rheumatic Diseases, Oregon Health & Science University, Portland, OR, United States; ^3^Seoul National University College of Medicine, Seoul, Republic of Korea; ^4^Mengchao Hepatobiliary Hospital, Fuzhou, China; ^5^First Affiliated Hospital of Zhengzhou University, Zhengzhou, China

**Keywords:** precision medicine, diagnosis, treatment, multi-dimension analysis, artificial intelligence

As editors of this Research Topic, it was our pleasure to review a wide range of fascinating articles in the field of multi-dimensional diagnosis and treatment strategies within precision medicine. In this editorial we summarize the main findings and perspectives detailed within each of the accepted articles.

The antibiotic teicoplanin is an important piece of our armory against bacteria that are resistant to many first-line treatments, including methicillin-resistant *Staphylococcus aureus* (MRSA). It has a long duration of action, however, to ensure efficacy, especially for bone and joint infections, a target “trough” systemic concentration is needed to be maintained. Ma et al.'s objective was to establish an optimal model to predict the teicoplanin trough concentrations by machine learning, and explain the feature importance in the prediction model using the game-theory based SHapley Additive exPlanation (SHAP) method. To do this, a retrospective study was performed on 279 therapeutic drug monitoring (TDM) measurements obtained from 192 patients who were treated with teicoplanin intravenously. Analysis revealed three algorithms with high *R*^2^ scores. These were selected to construct an ensemble which showed an absolute predictive accuracy of 83.93%, and relative predictive accuracy of 60.71%. Therefore, the authors demonstrated that a machine learning approach to predict the teicoplanin trough concentration was indeed possible.

Tacrolimus is a potent immunosuppressor used in many conditions including post-transplant kidney rejection. However, the narrow therapeutic index of tacrolimus and considerable response variability among individuals are challenges. Zhang et al. aimed to compare different machine learning and deep learning algorithms to establish individualized dose prediction models. The TabNet algorithm outperformed all other algorithms tested with the highest *R*^2^, the lowest error, and good performance of overestimated or underestimated dose percentage. This study illustrates that the TabNet model can be used predict dose and this technique is expected to be applied in future clinical practice.

Immune checkpoint inhibitors (ICI) have been applied in treating advanced hepatocellular carcinoma (aHCC) patients, but few patients exhibit stable and lasting responses. Lee et al. aimed to evaluate whether dissecting peripheral immune cell subsets by Mann-Whitney *U*-test and artificial intelligence (AI) algorithms could serve as predictive biomarkers of nivolumab treatment for aHCC. The disease control group carried significantly increased percentages of PD-L1^+^ monocytes, PD-L1^+^ CD8^+^ T cells, PD-L1^+^ CD8^+^ NKT cells, and decreased percentages of PD-L1^+^ CD8^+^ NKT cells as assessed by Mann-Whitney *U*-test. Using recursive feature elimination methodology, five featured subsets (CD4^+^ NKTreg, PD-1^+^ CD8^+^ T cells, PD-1^+^ CD8^+^ NKT cells, PD-L1^+^ CD8^+^ T cells, and PD-L1^+^ monocytes) were selected for AI training. Trained AI algorithms committed valuable AUC from 0.8417 to 0.875 to significantly separate disease control group from disease progression group, and SHAP value ranking also revealed PD-L1^+^ monocytes and PD-L1^+^ CD8^+^ T cells exclusively and significantly contributed to this discrimination. The authors therefore showed that integrally analyzing immune cell profiling with AI algorithms could serve as predictive biomarkers of ICI treatment.

Remaining with hepatocellular carcinoma (HCC), Huang et al. aimed to construct an epithelial mesenchymal transition (EMT)-related lncRNA signature for predicting the prognosis of HCC patients. Using Cox regression analysis and LASSO regression methodology the authors built an EMT-related lncRNAs risk signature based on the publicly available TCGA database. Twelve EMT-related lncRNAs were obtained to construct the prognostic risk signature, with patients in the high-risk group suffering worse overall survival (OS) than those in low-risk group. ROC curves and Cox regression analysis suggested the risk signature could predict HCC survival exactly and independently. The prognostic value of the risk model was confirmed in the testing and entire groups. Two specific predictive lncRNAs were highlighted (AC099850.3 and AC092171.2) and were found to be highly expressed in HCC cells and HCC tissues. This study showed that an EMT-related lncRNAs risk signature could identify patients with HCC, and accurately predict prognosis.

Diagnosis based on symptoms and presentation is often inadequate in explaining the underlying molecular-genetic abnormalities and individual genomic disparities. Yip et al. argue that current analytical trends rely too heavily on algorithms that recognize disease subtypes through gene expression, and that a gap exists in describing the extent of homogeneity within the heterogeneous subpopulation of different diseases, resulting in inaccurate diagnosis and subsequent management. Addressing these issues, the authors propose a framework, ReDisX, which introduces a unique classification system based on the patient's genomic signature. Here, Yip et al. apply this scalable machine learning algorithm and deployed to re-categorize patients with rheumatoid arthritis and coronary artery disease. Intriguingly, the results reveal heterogeneous subpopulations within a disease and homogenous subpopulations across different diseases. Therefore, the ReDisX framework may offer a novel strategy to redefine disease diagnosis through characterizing personalized genomic signatures.

Extramammary Paget's disease (EMPD) is a rare cutaneous neoplasm with distant metastases and a poor prognosis. This interesting case report authored by Yin et al., featured a 63-year-old male patient exhibiting stage IV primary EMPD with neuroendocrine differentiation, and harboring a somatic mutation in AMER1. After four cycles of Anlotinib (a VEGFR inhibitor) combined with Tislelizumab (an anti-PD1 monoclonal), the patient achieved partial response for the metastatic lesions according to mRECIST1.1 criteria. Total positron emission tomography and computed tomography (PET-CT) scans revealed a significant reduction in SUV from 18.9 to 5.3, and the serum CEA decreased to normal levels after the treatment regimen. However, the patient developed fractures of the fourth and fifth thoracic vertebrae during the treatment. Therefore, percutaneous vertebroplasty was performed, and the patient experienced severe postoperative pneumonia and died from pulmonary encephalopathy and respiratory failure in June 2021. The overall and progression-free survival of the patient after diagnosis were 9 and 8 months, respectively. During the systemic treatment, the patient suffered grade 1 rash in the back and thigh and grade 1 hypertension. Nevertheless, the combination treatment of anlotinib and tislelizumab had a favorable clinical outcome and provided a survival advantage, and, the authors argue, should be considered a therapeutic option for patients with AMER1-mutant metastatic EMPD.

It is clear from this “snapshot” of the field that this Research Topic provides, that predicative algorithms, machine learning and artificial intelligence hold great promise in interpreting multi-parametric data for clinical gain (1). These parameters are numerous, and growing both in terms of breadth and depth, and, with the community having implicitly agreed that disease complexity demands increased data, we have collectively passed the point of no return in requiring informatic help. However, we haven't yet settled on a set of parameters across multiple “omics” or analysis and/or interpretation systems for any specific disease. To overcome this, large population studies will be crucial in testing different parameter sets and analysis tools, and, within this space, it is reassuring to see moves away from classical Gaussian models toward more individualistic approaches, more reflective of disease heterogeneity.

Of course progress is often enhanced by commercial interest and these fields are no different. In Europe, AI driven pathology software is already in use in clinics, and there is tremendous investment in parameter collection and disease association and prediction; whether DNA, RNA, circulating DNA, immunophenotyping, exosomes, lncRNA, volatile organic compounds, biome, or glycans. The task will be fusing these commercial interests, discoveries and applications into a set of affordable and effective disease-specific models, that can sit inside existing financial and material healthcare infrastructure. The brutal reality check of what is genuinely achievable within the confounds of our existing healthcare systems will likely to be the determinant in which technologies and analytical tools will progress, and which will not.

This editorial highlights the array of different data and different methodologies currently being tested internationally; from long non-coding RNA to immune subtypes, from single to multi-disease, and from population based to game-theory approaches. It will be fascinating to watch the growth of this industry, the challenges it will face and its application into routine medical care over the next decade.

## Author contributions

RB and C-QC wrote the manuscript. SY, XL, and JZ reviewed the manuscript. All authors contributed to the article and approved the submitted version.
